# A Polyadenylation Factor Subunit Implicated in Regulating Oxidative Signaling in *Arabidopsis thaliana*

**DOI:** 10.1371/journal.pone.0002410

**Published:** 2008-06-11

**Authors:** Jingxian Zhang, Balasubramanyam Addepalli, Kil-Young Yun, Arthur G. Hunt, Ruqiang Xu, Suryadevara Rao, Qingshun Q. Li, Deane L. Falcone

**Affiliations:** 1 Kentucky Tobacco Research and Development Center, University of Kentucky, Lexington, Kentucky, United States of America; 2 Department of Plant and Soil Sciences, University of Kentucky, Lexington, Kentucky, United States of America; 3 Department of Botany, Miami University, Oxford, Ohio, United States of America; 4 Department of Biological Sciences, University of Massachusetts, Lowell, Massachusetts, United States of America; Massachusetts General Hospital, United States of America

## Abstract

**Background:**

Plants respond to many unfavorable environmental conditions via signaling mediated by altered levels of various reactive oxygen species (ROS). To gain additional insight into oxidative signaling responses, *Arabidopsis* mutants that exhibited tolerance to oxidative stress were isolated. We describe herein the isolation and characterization of one such mutant, *oxt6*.

**Methodology/Principal Findings:**

The *oxt6* mutation is due to the disruption of a complex gene (At1g30460) that encodes the *Arabidopsis* ortholog of the 30-kD subunit of the cleavage and polyadenylation specificity factor (CPSF30) as well as a larger, related 65-kD protein. Expression of mRNAs encoding *Arabidopsis* CPSF30 alone was able to restore wild-type growth and stress susceptibility to the *oxt6* mutant. Transcriptional profiling and single gene expression studies show elevated constitutive expression of a subset of genes that encode proteins containing thioredoxin- and glutaredoxin- related domains in the *oxt6* mutant, suggesting that stress can be ameliorated by these gene classes. Bulk poly(A) tail length was not seemingly affected in the *oxt6* mutant, but poly(A) site selection was different, indicating a subtle effect on polyadenylation in the mutant.

**Conclusions/Significance:**

These results implicate the *Arabidopsis* CPSF30 protein in the posttranscriptional control of the responses of plants to stress, and in particular to the expression of a set of genes that suffices to confer tolerance to oxidative stress.

## Introduction

Plants encounter a broad range of challenges in the natural environment. Adverse conditions brought about by excesses of light or temperature, drought, salinity, atmospheric pollutants such as ozone, or soil-borne pollutants such as heavy metals, can all threaten plant health. Critical for their survival is the ability of plants to perceive and rapidly respond to the stresses caused by such ever-changing conditions. Characteristic responses to most of these conditions are alterations in the cellular level of reactive oxygen species (ROS) [1]–[3]. The ensuing oxidative stress produced by severe increases in ROS can be deleterious to plants, as ROS such as the superoxide radical, hydrogen peroxide, or the hydroxyl radical are able to damage most cellular macromolecules directly, and ultimately lead to cell death [2], [4], [5]. On the other hand, ROS are produced continuously at basal levels as a consequence of a wide range of common metabolic processes. This, together with the transient increases in ROS concentrations when subjected to unfavorable conditions, make ROS well suited to be key regulatory molecules of plant responses to stress [1], [3], [6] as well as more general signaling molecules for a variety of plant processes such as stomatal closure, cell expansion, root gravitropism, and aspects of development [7], [8].

The recognition of ROS signaling in several plant processes has led to considerable interest in studying the expression of ROS regulated genes. Exposure to ROS triggers a sizeable gene expression program [9], [10], including the activation of a set of genes encoding a characteristic array of detoxifying enzymes [11]–[13]. This increased gene expression probably involves one or more signaling pathways, modulation of which results in changes in transcriptional activity of a variety of genes encoding detoxifying enzymes. Components of several regulatory systems have been implicated in responses to ROS. One of these are protein kinases (MAPKs); ROS or treatments that increase ROS activate a number of plant MAPKs, and ROS may act through activation of an upstream kinase, OXI1 [14], which has recently been proposed to also enable lipid-derived signals to be integrated via an oxidative signaling module, PDK1-OXI1 [15]. Additionally, the transcription factor, ZAT12, a C_2_H_2_-type zinc finger protein, has been implicated in early steps in the response of *Arabidopsis* to ROS and to abiotic stresses that are associated with ROS signaling [16], [17]. Transcription of ZAT12 is itself upregulated in response to ROS, as well as being required for the ROS-regulated expression of at least one other stress-associated transcription factor, ZAT7, another C_2_H_2_-type zinc finger protein [17]. ZAT12, in turn appears to be regulated by HSF21, a redox-sensitive transcription factor [18]. An additional ZAT family transcription factor, ZAT10, has recently been shown to be involved in high-light acclimation of leaves, a process also tied to ROS signaling [19], [20]. Among the consequences of exposure to environmental stress signals, including ROS, is a rapid elevation of intracellular calcium levels [21]–[24]. These observations, along with the reported calcium-dependent activation of the H_2_O_2_ detoxifying enzyme catalase by calmodulin [25], suggest that calcium-mediated signaling is important in response to ROS. The mechanisms by which these systems cooperate or intersect, how their activities are affected by other stress- and pathogen- related signaling pathways, and how the initial stress state is sensed remain to be worked out.

To gain further understanding of the processes plants use to perceive and respond to stress, we applied a genetic screen based on a whole-seedling phenotype to isolate lines that exhibit enhanced growth in the presence of conditions that elicit oxidative stress. We present here the characterization of an *Arabidopsis* mutant that tolerates oxidative stress, *oxt6*. The *oxt6* mutation was caused by a T-DNA insertion into a gene encoding a polyadenylation factor subunit homolog, CPSF30. The properties of this mutant are consistent with a model whereby stress signals are linked to downstream stress tolerance responses that are modulated by RNA processing.

## Results

### Isolation of an Arabidopsis oxidative stress-tolerant mutant, Oxt6

To isolate mutants of *Arabidopsis thaliana* that possess enhanced tolerance to oxidative stress, a genetic screen based on a whole seedling phenotype was developed. This screen employed 3-amino-1, 2, 4-triazole (AT), a catalase inhibitor that elevates intracellular H_2_O_2_ in cultured *Arabidopsis* cells [26]. Since addition of AT to cells also leads to increased glutathione levels [26], and since higher glutathione concentrations are expected to ameliorate the increased H_2_O_2_ levels caused by AT treatment, buthionine S,R-sulfoximine (BSO), an inhibitor of glutathione synthesis [27], was also included to insure the stress-inducing conditions. A range of AT and BSO concentrations were tested to find a combination that caused uniform growth inhibition without killing the plants. AT and BSO at 2.0 μM and 400 μM, respectively, resulted in a uniform inhibition of root growth when roots penetrated the agar medium, but did not result in immediate death of wild-type seedlings (Sukrong et al., unpublished data). These conditions were used to screen for mutants that displayed longer primary roots after growth under the imposed stress.

In a population of ∼10,000 independent T-DNA mutagenized *Arabidopsis* lines, 326 gave root lengths at least 130% longer than wild type and 35 of this group showed heritability for elongated roots. These 35 lines were examined for tolerance to methyl viologen (MV). MV generates ROS via the production of superoxide radicals primarily at photosystem I, a mechanism distinct from AT and BSO; mutants resistant to both AT+BSO and MV are expected to be affected in general responses to ROS, as opposed to pathways specific for either metabolism related to these chemicals or to the specific oxidative species involved. Several lines, designated *oxt* for *ox*idative stress *t*olerant, displayed reduced sensitivity to AT and BSO as well as to MV. One such line, *oxt6*, was selected for further study.

Genetic analysis was conducted by backcrossing *oxt6* to the wild type and scoring the resulting F_1_ progeny for either tolerance to AT plus BSO, or for the distinctive dwarf phenotype seen in the mutant plants when grown in soil (see below). All F_1_ heterozygotes exhibited wild-type phenotypes under both stress and soil-grown conditions. Selfed F_2_ progeny of individuals from these backcrosses showed a segregation ratio close to 3∶1 (447 long roots∶140 short roots, χ^2^ = 0.4; P>0.05) for tolerance to MV, indicating that *oxt6* is a recessive mutation that confers tolerance to oxidative stress. PCR analysis confirmed that T-DNA was present in all MV-tolerant plants. MV-sensitive individuals that arose from the same backcross segregated for the presence of the T-DNA in a ratio of 68∶30, close to an expected 2∶1 ratio (χ^2^ = 0.33, P>0.05), indicating that *oxt6* contained a single T-DNA insertion that was very likely linked to the mutant phenotype.

### Oxidative stress tolerance in the oxt6 mutant

**Figure 1:**
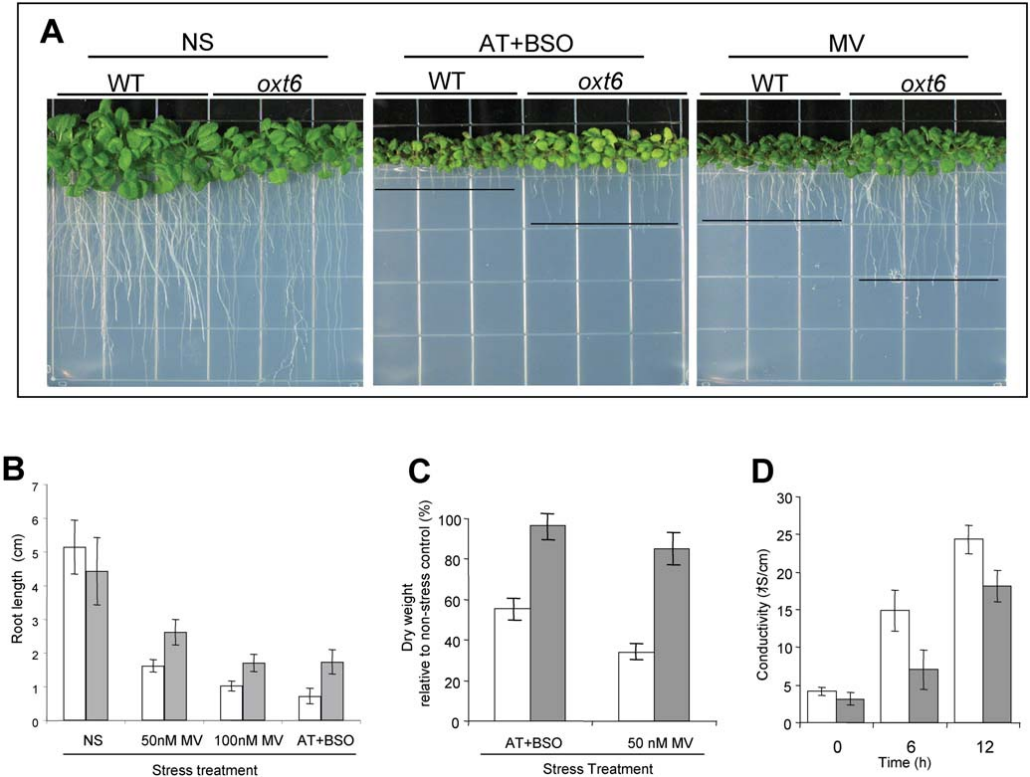
Phenotypes of the *oxt6* mutant. (A) Images of wild-type and *oxt6* plants growing on MS+1% sucrose agar medium only [non-stress (NS)] and under stress conditions induced by 2.0 μM aminotriazole +0.4 mM buthionine sulfoximine (AT+BSO) and 50 nM methyl viologen (MV). (B) and (C). Effects of AT+BSO and MV treatments on root length (B) and dry mass accumulation (C) in the wild type and *oxt6* mutants. Dry weight data are plotted as a percentage of the accumulation of each line under non-stressed conditions. (D) Reduced cell damage in isolated leaf discs in the presence of methyl viologen as measured by ion leakage. Leaf discs were incubated in 2.0 μM methyl viologen at 22°C at 80 μmol m^−2 ^s^−1^ and ion leakage determined as described in Materials and Methods. (B) through (D), white bars, wild type, gray bars, *oxt6*.

**Figure 2:**
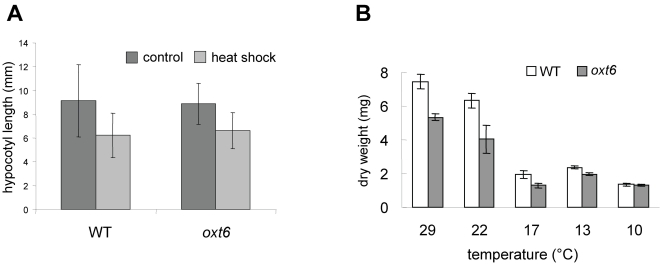
Responses of the *oxt6* mutant to high and low temperature. (A) Responses of plants to elevated temperatures. Young seedlings were exposed to 40°C for a brief period of time and hypocotyls elongation measured after returning the plants to room temperature (see Methods). (B) Seven-d-old plants were grown on MS+sucrose medium at 22°C and then grown at the indicated temperatures for14 days, when the dry mass accumulation was determined. Samples in both plots are as indicated in the legend in the insets.

**Figure 3:**
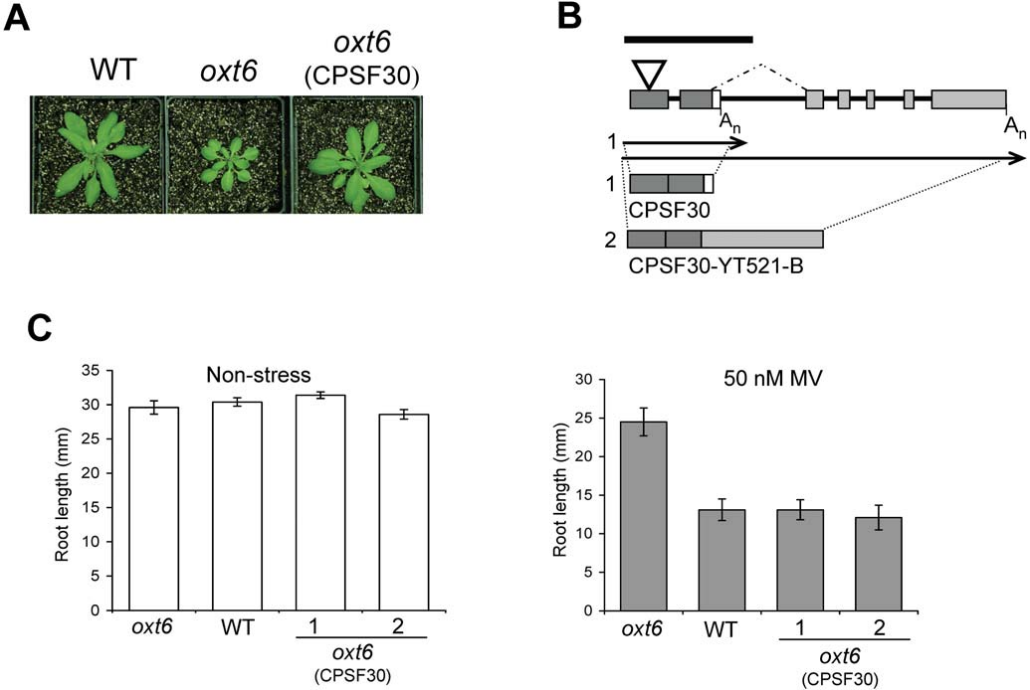
Complementation of the mutant phenotype by the AtCPSF30 coding region. (A) Photograph of representative plants grown in soil under unstressed conditions. (B) Schematic representation of the *OXT6* gene structure. Dark gray boxes indicate the exons present in the smaller of the two transcripts encoded by this gene, while light gray boxes represent additional exons present in the larger of the two *OXT6*-encoded RNAs. Note that the sequence represented by the small white box is absent in the larger RNA. The structures of the spliced RNAs (“1” and “2”) are depicted beneath the illustration of the genomic DNA. The solid line above the illustration of the genomic DNA indicates the extent of the gene that was expressed for complementation studies (note that the promoter sequences are not shown in the illustration). The polyadenylation site within the second intron that is utilized for production of the shorter transcript is denoted as A_n_, and the splice junctions that define the second intron linked together above the depiction of the genomic DNA. (C) Root lengths of wild-type, *oxt6* mutants, and *oxt6*:AtCPSF30 plants after exposure to 50 nM MV.

*Oxt6* plants were somewhat smaller than wild type when grown under non-stress conditions in both MS agar medium (Figure 1A and 2B) and soil (Figure 3A). The relative growth rate of wild type calculated from day 8 to day 14 was 0.323 mg^−1^d^−1^ while the *oxt6* relative growth rate over this same interval was 0.213 mg^−1^d^−1^, indicating that the wild type grew 1.5-times faster than *oxt6* at earlier stages. After 14 days however, the wild-type and *oxt6* growth rates were similar, such that the *oxt6* rosette leaves attained a mean diameter of about 0.7 times that of the wild-type rosette before bolting. The *oxt6* mutant also exhibited a modest delay in development under non-stress conditions in long day lengths, as reflected by the number of leaves within the rosette at the time of bolting. Prior to bolting, wild-type rosettes had 11.9±0.8 leaves, while rosettes on *oxt6* mutant plants had 13.8±0.6 leaves (n = 10 plants each; ±SE).

After 14 days of growth on agar media, untreated *oxt6* mutants had root lengths that were about 85% of the wild type (Figure 1B). In contrast, the root lengths of *oxt6* plants grown in the presence of MV or AT+BSO were 150%–250% of the treated control plants (Figure 1B). In terms of the inhibition of root growth, the MV and AT+BSO treatments inhibited wild-type root growth by between 70% and 90%, respectively, while the same treatments reduced *oxt6* root growth by 40%–60% (Figure 1B). After 14 days of growth on agar plates, AT+BSO-treated *oxt6* plants accumulated about 95% of the aerial dry mass as did untreated *oxt6* plants, while MV-treated *oxt6* plants accumulated about 90% of the aerial dry mass that untreated *oxt6* plants did (Figure 1C). In contrast, the growth of wild-type plants was inhibited by about 45% and 70%, respectively, by these treatments (Figure 1C).

Cellular ion leakage in leaf explants can be used as a means to quantify damage induced by oxidative stress, and thereby provide an assessment of stress tolerance independent of growth characteristics. Untreated wild-type and *oxt6* plants showed a similar (low) degree of ion leakage, assessed as the conductivity of the medium in which the leaf explants were incubated (Fig. 1D). After 6 hr in the presence of MV, ion leakage in the mutant was some 48% of that seen in the wild-type (Fig. 1D), indicative of a significant reduction in MV-induced cellular damage. After 12 hours, ion leakage in the mutant was some 70% of that seen in the wild-type (Figure 1D). This experiment demonstrates a considerable protection against the damaging effects of MV in the mutant.

Reactive oxygen signaling is associated with numerous biotic and abiotic stresses. Because of these interrelationships, it is possible that the tolerance to chemically-induced oxidative stress exhibited by the *oxt6* mutant is due to other changes in responses to stresses that may include constitutive conditioning of plants to tolerate ROS. Alternatively, tolerance to ROS may manifest itself as changes in responses to stresses (such as elevated or low temperatures) that are accompanied by production of ROS. To test this, the responses of the mutant to high temperature treatment (Figure 2A) and to growth at high and low temperatures (Figure 2B) were examined. As shown, the responses of the *oxt6* mutant to these treatments were very similar to those of its wild-type parent. Thus, the *oxt6* mutant does not display a general or global alteration in responses to abiotic stress, but rather a more limited change in the susceptibility just to ROS elicited, for example, with MV.

### Molecular characterization and complementation of the oxt6 mutant

**Figure 4:**
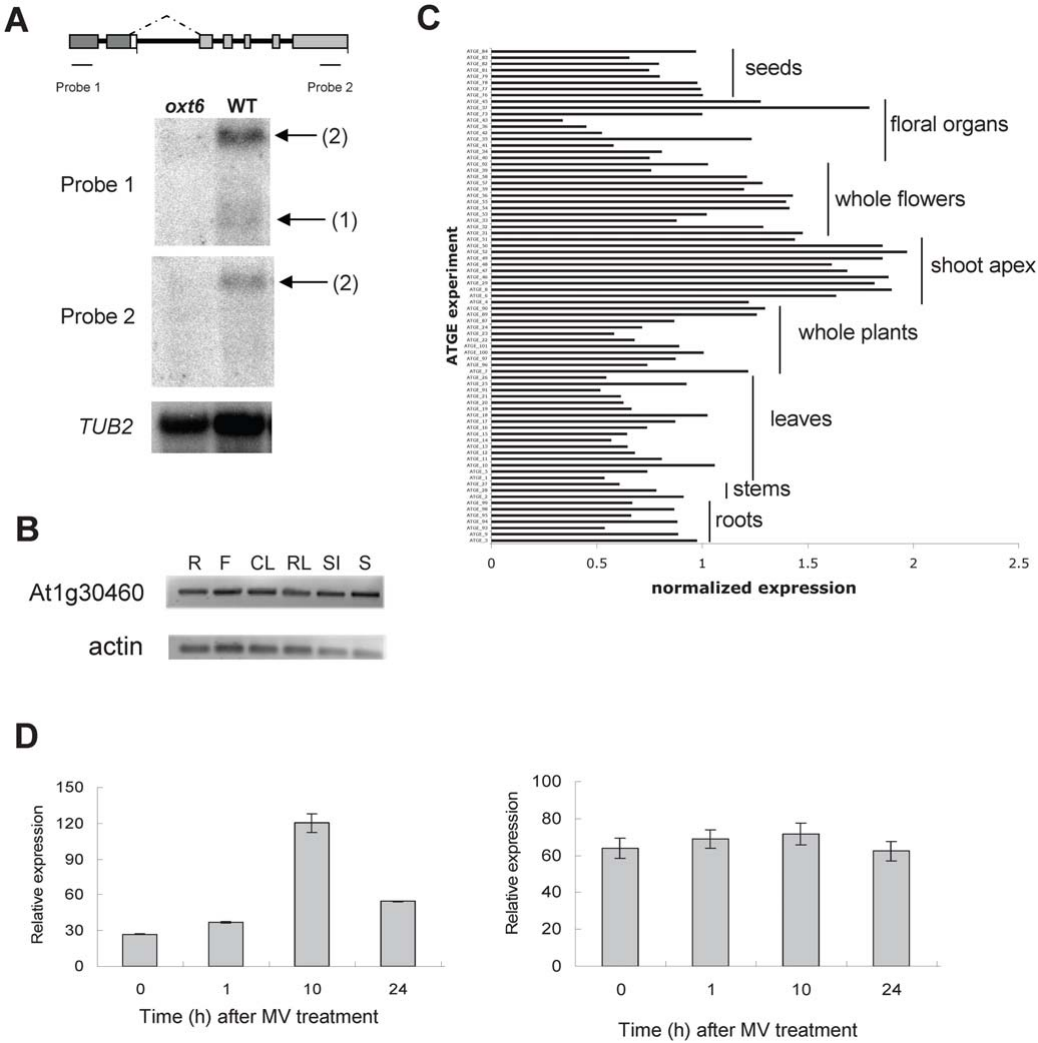
*OXT6* gene expression profile. (A) RNA blot analysis of the expression of the *OXT6* gene in the *oxt6* mutant and wild-type (WT) plants. The structure of the *OXT6* gene is shown at the top, with the positions of the probes (“Probe 1” and “probe 2”) used for the RNA blotting highlighted. The expression of the *Arabidopsis* tubulin gene is shown beneath the two *OXT6* gene probe blots. “(1)” and “(2)” indicate transcripts sizes as those depicted in the schematic in Figure 3B. (B) RT-PCR analysis of *OXT6* gene expression in different plant tissues. Total RNA was isolated from root (R), flower (F), cauline leaf (CL), rosette leaf (RL), silique (Si), and stem tissues (S) from mature (25-day-old) plants and gene primers specific to the small (AtCPSF30) transcript were used following reverse transcription as described in Materials and Methods. (C) Meta-analysis of the expression of the *OXT6* gene. Expression data for this gene (probe 261798_at) for the developmental series available from NASC was extracted and the normalized expression values plotted as shown; this probe set recognizes just the larger of the two *OXT6*-encoded RNAs. Developmental stages for the sets of samples are indicated on the plot. The full dataset is provided in Supporting Table S5. (D) RT-PCR analysis of the expression of the small (left panel) and large (right panel) mRNAs in wild type plants exposed to methyl viologen (MV) for 1, 10 and 24 hr.

Using PCR primers specific for the modified T-DNA used to mutagenize the *Arabidopsis* population, the genomic position of the *oxt6* mutation was found to lie 147 bp downstream of the translation initiation codon within the first exon of a gene, At1g30460, located on *Arabidopsis* chromosome 1 (Figure 3B). This gene encodes two mRNAs (Figure 4A) and, as shown in a previous study, two polypeptides, owing to alternative poly(A) site use [28]. The smaller of these is similar to yeast and mammalian polyadenylation factor subunits (Yth1p and the 30 kD subunit of cleavage and polyadenylation specificity factor, or CPSF30, respectively; [28]), while the larger polypeptide consists of the CFSP30-related domain fused to a second domain that is related to a mammalian splicing factor-related protein (YT521-B; [29]. The *oxt6* mutant lacks both polypeptides, as shown in previous work [28], as well as their encoding mRNAs (Figure 4A;). The *OXT6* gene appears to be expressed in all tissues, judging from the results of RT/PCR analysis (Figure 4B) and from perusal of public domain microarray data (Figure 4C) (Supporting table S5). The latter analysis shows that expression of the At1g30460 gene varies at most by about 4-fold over the range of samples analyzed, with the highest expression in the shoot apex and lowest in stamens. Interestingly, the smaller but not larger of the two *OXT6*-encoded mRNAs was transiently up-regulated by exposure to MV (Figure 4D).

To better understand the contributions of the two mRNAs to the *oxt6* phenotype, genomic sequences extending from ca. 2000 bp upstream of the initiation codon of the *OXT6* coding region(s) to 500 bp downstream of the termination codon of the smaller mRNA were introduced into the *oxt6* mutant. The resulting plants (*oxt6*/CPSF30) did not display the dwarf phenotype that was apparent in the *oxt6* mutant (Figure 3A). Moreover, they were restored in terms of their sensitivity to oxidative stresses. Thus, *oxt6*/CPSF30 plants showed the same 70–90% reduction in root growth in the presence of AT+BSO (not shown) or MV than the wild-type plants did (Figure 3C). Dry mass accumulation in *oxt6*/AtCPSF30 was likewise reduced by some 60% by these treatments (not shown). These data indicate that the smaller of the two *OXT6*-derived mRNAs, and the AtCPSF30 protein, is sufficient to restore a wild-type stress-sensitive phenotype to the *oxt6* mutant.

### Transcriptional profiling – ROS-related genes that are altered in a CPSF30-dependent fashion

To better understand the link between AtCPSF30 and the *oxt* phenotype, transcriptional profiling using the Affymetrix ATH1 Genome Array was conducted. For this, genes whose expression correlated with the ROS-tolerant phenotype were identified; thus, genes whose expression was significantly different in the *oxt6* mutant compared with both the wild-type and complemented plants, but whose expression was not accordingly different when the wild-type and complemented plants were compared, were identified. This exercise yielded 353 probes whose expression correlated with the stress-tolerant phenotype. Because of some ambiguity in the probe-gene correspondence, this set of 353 probes identified 362 genes (Supporting Table S1). A number of enzyme classes participate in the control of reactive oxygen species; these include ascorbate peroxidases, catalases, superoxide dismutases, glutathione-S-transferases, peroxiredoxins, glutaredoxins, and thioredoxins [3], [12], [13], [30], [31]. Among the set of 362 genes identified in the transcriptional profiling, only two of these classes were substantially represented (Table 1). Specifically, the expression of nine genes encoding proteins containing either glutaredoxin- or thioredoxin- related domains were up-regulated by two or more fold in the mutant compared with the wild-type or with the complemented lines.

**Table 1:** Thioredoxin- and glutaredoxin- related genes whose expression is two-fold or more greater in the *oxt6* mutant than the wild-type.

Locus Identifier	Annotation	wt/mut	comp/mut	wt/comp
AT1G07960	protein disulfide isomerase-like (PDIL)	0.38 (0.18)	0.42	0.90
AT1G19730	ATTRX4 (thioredoxin H-type 4)	0.44	0.14	3.06
AT3G25580	thioredoxin-related	0.22 (0.31)	0.41	0.53
AT3G62950	glutaredoxin family protein	0.30	0.40	0.76
AT4G15660	glutaredoxin family protein	0.30	0.24	1.23
AT5G06430	thioredoxin-related	0.49	0.23	2.12
AT5G06690	thioredoxin family protein	0.39 (0.96)	0.40	1.00
AT5G18120	protein disulfide isomerase-like (PDIL)	0.29	0.44	0.66
AT5G63030	glutaredoxin, putative	0.41	0.34	1.22

The columns labeled “wt/mut”, “comp/mut”, and “wt/comp” denote the respective expression ratios for these genes. Values in parentheses represent ratios derived from RNA blot analysis.

wt – wild-type parent of *oxt6*; mut – the *oxt6* mutant; comp – *oxt6*-derived lines that express a gene encoding just the smaller (CPSF30) of the two At1g30460-encoded proteins.

The result obtained from identifying genes whose expression is significantly correlated with the ROS-tolerant phenotype was corroborated by a gene-by-gene analysis of expression in the various lines (Supporting Table S4). Thus, 20 (of 59) thioredoxin-related genes and 12 (of 23) glutaredoxin-related genes showed variation of expression that correlated with ROS tolerance (consisting of elevated expression in the mutant compared with either the wild-type or complemented plants, and little difference or elevated expression in the wild-type compared with the complemented plants). Of the set of 26 genes encoding detoxifying activities (ascorbate reductases and peroxidases, catalases, superoxide dismutases, and peroxiredoxins), only one (FSD2, corresponding to At5g51100) had an expression profile that correlated with ROS tolerance (Supporting Table S2).

**Figure 5:**
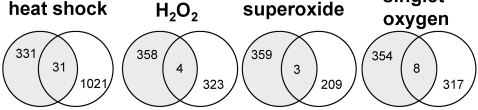
Venn diagrams of genes impacted by AtCPSF30 and various stresses. Shown are the commonalities of genes dependent on the presence of AtCPSF30 and those genes that are responsive to heat shock, hydrogen peroxide treatment (H_2_O_2_), superoxide treatment, and increased levels of ozone (singlet oxygen). Shaded circles represent genes whose expression is significantly affected by the absence of AtCPSF30, while white circles denote genes affected by the other indicated treatments. The genes represented in each set of overlaps are listed in Table S7.

Other reports describing transcription profiling of stress responses in *Arabidopsis* have been published, and experiments of time courses of the responses of *Arabidopsis* to numerous stresses are available in public microarray data repositories. To assess the relationships between these treatments and the phenotype of the *oxt6* mutant, the lists of genes most responsive in other studies were compared with the AtCPSF30-specific genes identified in this study. As indicated in Figure 5, four, three, and eight genes, respectively, were shared between the AtCPSF30-specific list and the lists of genes most responsive to elevated hydrogen peroxide, superoxide, and singlet oxygen species, respectively [11]. In addition, the set of genes that changed in an AtCPSF30-dependent manner contained 31 members (or 8.6%) that also were strongly-induced by heat shock [32] (Supporting Table S2, S7). These comparisons do not lend strong support to the hypothesis that the *oxt6* mutant affects gene sets that also are responsive to ROS, but they do indicate a very modest commonality between heat shock and the effects of the *oxt6* mutation. The commonality seen with heat shock genes, however, is not manifest as increased tolerance of elevated temperatures (Figure 2A, B).

### Altered poly(A) site choice in the oxt6 mutant

**Figure 6:**
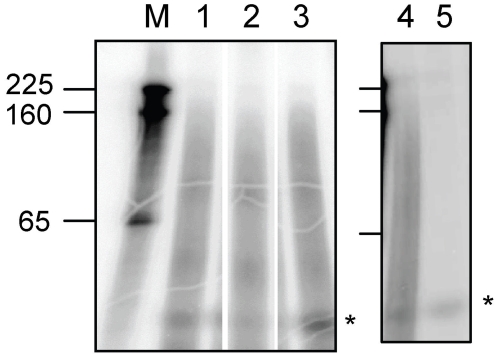
Bulk poly(A) length is not affected in the *oxt6* mutant. Results of samples obtained from the wild-type (lane 1), *oxt6* mutant (lane 2), and complemented plants (lane 3) are shown. Samples treated with oligo-dT in the absence (lane 4) or presence (lane 5) of RNAse H are shown on the right-hand panel. A small RNA that is resistant to the RNAse A+T1 treatment is denoted with *. RNA size standards are in lane M, and the sizes indicated on the left.

Several lines of evidence indicate that AtCPSF30 is a polyadenylation factor subunit [28], [33]–[35]. Thus, it may be that the length of poly(A) tails on mRNAs in the *oxt6* mutant may be different from those in the wild-type or complemented plants. This was tested by directly measuring the length distribution of bulk poly(A) in the three lines. As shown in Figure 6, poly(A) lengths ranged from very short to somewhere between 160 and 200 nts in the wild-type, mutant, and complemented plants, with no obvious differences between the three samples. Treatment with RNAse H+oligo-dT eliminated the labeled products (Figure 6, lane 5 and unpublished observations), indicating that the observed products were authentic poly(A). These results thus indicate that the disruption of the At1g30460 gene does not have a dramatic effect on poly(A) length.

**Figure 7:**
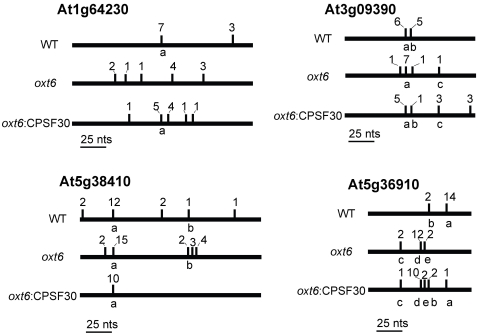
3′ end choice is different in the *oxt6* mutant and in complemented plants. The results of sequencing of collections of 3′-RACE clones are illustrated here; each vertical tic represents a distinct 3′ end. Lower case letters denote 3′ ends that are the same in the various collections. Numbers above the line denote the numbers of independent clones with 3′ ends at the corresponding site. The size of a 25 nt increment is shown beneath each depiction. The translation termination codons for these genes were 86 (At1g64230), 186 (At3g09390), 60 nts (At5g38410), and 158 (At5g36910) upstream, respectively, from the 5′ extremities of the sequences illustrated here and provided in Table S3.

The *Arabidopsis* CPSF30 protein has been suggested to be directly involved in the processing of the pre-mRNA prior to polyadenylation [33]. Thus a consequence of a deficit of AtCPSF30 might be an alteration of poly(A) site choice in particular genes. This hypothesis was tested in a small set of genes; three of these genes were not significantly different in terms of their expression in the three backgrounds, whereas one (At5g36910) was up-regulated by some 10–15 fold in the mutant compared with both the wild-type and complemented plants (not shown). The results of this experiment are summarized in Figure 7, and the collection of sequences listed in Supporting Table S3. For all four genes, the sites seen in the wild-type were identical in position and approximate abundance to sequences that may be found in EST databases (not shown), indicating that the approach is a valid and accurate estimation of poly(A) site profiles in these four genes.

The poly(A) site profiles were different in the three sets of plants. For At1g64230, neither of the two sites used in the wild-type were seen in the *oxt6* mutant. Much of the wild-type usage was restored in the complemented plants, but other sites not seen in either the mutant or wild-type were also selected. For At5g36910, the wild-type 3′ end profile was not seen in the mutant. However, some wild-type poly(A) site usage was seen in the complemented plants, although some 3′ ends that corresponded to those seen in the mutant were also apparent. For At3g09390, a majority of the 3′ ends in the mutant and complemented plants corresponded to the sole site seen in the wild-type. In the mutant and complemented plants, however, additional sites were seen, some of which were unique to either mutant or complemented plants. For At5g38410, the predominant wild-type site was also the predominant site used in the mutant lines, and was used exclusively in the complemented plants. Increased usage of an additional wild-type site was seen in the mutant. However, there were several sites that were unique to the wild-type or mutant. Taken together, these results show that poly(A) site choice is different in the *oxt6* mutant compared with the wild-type and complemented plants, and indicate that alternative poly(A) site choice is a consequence of the absence (or not) of AtCPSF30.

## Discussion

### The involvement of CPSF30 in responses to ROS

The signaling events involved in the responses of plants to oxidative stress include calcium fluxes, protein kinase cascades, and transcription factors [1], [4], [14], [17], [18], [21], [23], [25], [30], [36]; these combine to promote increased production of enzymes that reduce reactive oxygen species and ameliorate the effects of ROS on cellular processes [3], [4], [11], [12], [25], [30], [31], [37]–[39]. The multiplicity of inducing agents (superoxide, peroxide, singlet oxygen, and hydroxyl radicals) along with the diversity in cellular locations for the various systems (being located in the cytoplasm, chloroplast, peroxisome, and mitochondria) afford a large variety of both signaling mechanisms and enzymatic pathways to minimize the potential damage due to exposure to ROS. The *oxt6* mutant reveals an additional, as yet uncharacterized, route by which plants may respond to ROS signals. Most genes that encode detoxifying mechanisms are relatively unaffected by the *oxt6* mutation, but a number of genes encoding thioredoxins and glutaredoxins are expressed at a higher level in the mutant. This observation suggests that modified expression of a relatively small subset of ROS-associated or -induced genes may suffice for a degree of tolerance to ROS. Moreover, the relatively specific (in terms of ROS-associated genes) effects of AtCPSF30 suggest that many members of the thioredoxin and glutaredoxin gene families may be subject to control by signaling pathways and environmental cues that are apart from those described to date.

**Figure 8:**
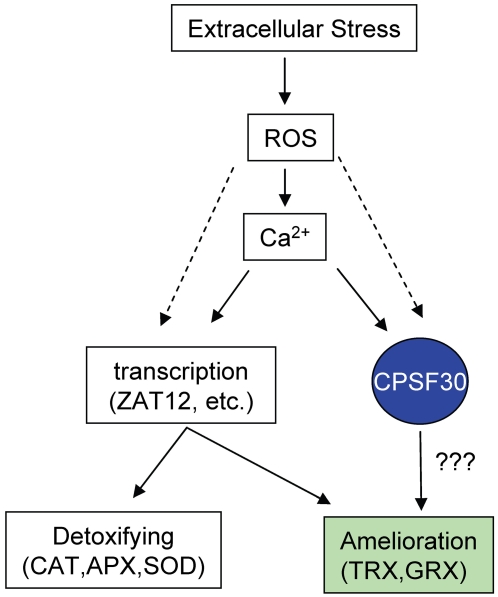
Working model showing the relationship between AtCPSF30 and reactive oxygen signaling in stress responses. Forms of extracellular stress that result in alterations in reactive oxygen species (ROS) cause rapid increases in cellular Ca^++^. “???” denotes as yet hypothetical steps between the consequences of inhibition of AtCPSF30 activity and induction of the genes (“TRX, GRX”) implicated in this study as being responsible for tolerance to MV-induced stress.

The link between AtCPSF30 and tolerance to oxidative stress is at first glance not apparent. One might hypothesize that AtCPSF30 directly affects the expression of a “master” regulator whose suite of clients includes those genes listed in Table 1. Alternatively, the stress-tolerant phenotype in the *oxt6* mutant might be a secondary consequence of a different primary effect, owing to changes in expression of genes somewhat removed from direct ROS responses. In either case, it is likely that AtCPSF30 plays a role in ROS-regulated gene expression. This follows from the recollection that AtCPSF30 is a calmodulin-binding protein, and its RNA-binding activity is inhibited by calmodulin in a calcium-dependent manner [28]. Rapid increases in intracellular Ca^2+^ concentrations are among the first events that occur after exposure of plants to oxidative stresses [22]–[24]. The increase in Ca^2+^ that accompanies the onset of stress would be expected to result in an inhibition of RNA binding by AtCPSF30. This would in turn mimic the situation seen in the *oxt6* mutant, triggering the gene expression program that includes increased production of thioredoxin- and glutaredoxin- related proteins. Thus, AtCPSF30 might be envisioned to be positioned relatively early in response to oxidative signals, along with a variety of other cellular components that possess calcium/calmodulin domains and are directly modulated by Ca^2+^ signals (Figure 8).

Other aspects of nuclear RNA processing have recently been implicated in the responses of plants to various abiotic stresses. For example, plants homozygous for one mutant allele (*los4-2)* of the *Arabidopsis LOS4* gene that encodes an RNA helicase involved in export of RNA from the nucleus to the cytoplasm are tolerant to cold and freezing stresses but more sensitive than the wild-type to heat shock [40]. Mutants homozygous for another allele of this gene, *los4-1*, are more sensitive to chilling and freezing stress [41]. In these two instances, the responses to chilling and freezing stress reflect the mRNA export characteristics of plants grown at low temperatures. Mutants in another component of the RNA export machinery, AtNUP160 (the *Arabidopsis* ortholog of the NUP160 subunit of the nucleoporin NUP107-160 subcomplex), are also sensitive to chilling and freezing stress [42]. Thus, RNA export would seem to be a crucial determinant of tolerance to low temperatures in *Arabidopsis*.

Loss-of-function mutations in another gene whose product is involved in nuclear RNA metabolism, *STABILIZED1*, also have differing effects on responses of plants to abiotic stresses [43]. In this case, mutants in this gene were more sensitive than the wild-type to ABA, cold stress, and LiCl, but were not affected in their responses to NaCl. Interestingly, these mutants seemed more tolerant to osmotic stress imposed by growth on mannitol. Thus, a theme that is reiterated by the *oxt6* mutant is apparent – loss of function of RNA processing enzymes appears to lead to differing but somewhat specific or focused effects on abiotic stress responses. Together, these studies insinuate RNA processing into the network of regulatory interactions involving reactive oxygen signaling in abiotic stress responses.

### AtCPSF30 and polyadenylation in plants

*OXT6* is the only *Arabidopsis* gene that encodes an obvious CPSF30/Yth1p homologue [28]. The smaller of the two *OXT6*-encoded proteins (AtCPSF30) is the CPSF30 ortholog; this protein is present in the nucleus and is the smaller of the two and resides in a complex with at least one other polyadenylation factor subunit, AtCPSF100 [28]. AtCPSF30 also interacts physically with an *Arabidopsis* ortholog of Fip1 [34]. AtCPSF30 has recently been reported to be an endonuclease, an activity that is inhibited by its association with the Fip1 ortholog [33]. These characteristics are consistent with a role for AtCPSF30 in the processing that precedes poly(A) addition. The results described in Figure 7 of this report buttress these other observations, in that they show a clear effect of a deficit in AtCPSF30 on poly(A) site choice; such a result is to be expected of a gene that encodes a processing endonuclease.

In yeast, Yth1p is an essential protein [44], [45], and it is to be expected that the same function would likewise be essential in plants. That the *OXT6* gene is nonessential is thus surprising. The reasons for the discrepancy between the yeast system and *Arabidopsis* are not known, but a number of interesting possibilities merit discussion. The *OXT6* gene may not be essential due to the presence in *Arabidopsis* of other proteins that function as does CPSF30 in mRNA 3′-end formation. While BLAST searches do not yield obvious candidates for such hypothetical proteins, *Arabidopsis* does possess a large family of CCCH zinc finger proteins [28], one or more of which may be able to replace AtCPSF30 in mRNA 3′ end formation. Moreover, there exists a possibility that other proteins, unrelated at the amino acid sequence (or even motif organization) level, may be able to provide the activity of CPSF30 in plants. One likely candidate is the *Arabidopsis* ortholog of CPSF73, which in mammals has been suggested to be a processing endonuclease in the polyadenylation reaction [46], [47].

Variation in poly(A) site profiles was seen in the complemented plants as well as the *oxt6* mutant (Figure 7, Supporting Table S3). However, each of the three lines studied –wild type, *oxt6* mutant, and complemented plants expressing just the smaller of the two At1g30460-encoded mRNAs –possessed distinctive profiles. The absence of complete restoration of wild-type poly(A) site choice in the complemented plants implicates both AtCPSF30 and AtCPSF30-YT521B in mRNA 3′ end formation. This in turn adds the possibility of additional control to the process. The YT521B domain that is present in the larger polypeptide is also found in a family of *Arabidopsis* proteins that bind to a protein kinase that may function in concert with calcineurin [48]. The significance of this association is not clear, but the link between calcineurin, the calcineurin B-like interacting protein kinase (CIPK1), and the YT521B domain raises the possibility of regulation of AtCPSF30-YT521B function via calcineurin. This would provide a link apart from calmodulin between calcium and mRNA 3′ end formation.

## Materials and Methods

### Mutant isolation

Seeds of *Arabidopsis thaliana* Columbia (Col-0) mutagenized by T-DNA (pROK2) insertion were obtained from the *Arabidopsis* Biological Resource Center (Ohio State University). Seedlings were germinated and grown in growth chambers set at 22 °C under continuous light (60–80 μmol m^−2^ s^−1^) on vertical plates containing 1% agar-solidified Murashige and Skoog (MS) mineral salts, 1% (w/v) sucrose and 0.5 mM MES. MS medium was supplemented with 2.0 μM 3-amino-1, 2, 4- triazole (AT) and 400 μM buthionine S,R sulfoximine (BSO) to conduct the primary screen. For the primary screen, 14-day-old seedlings displaying decreased sensitivity to the AT and BSO-induced stress conditions were scored by elongated roots, and transferred to soil to set seed. For the secondary screen and for follow-up phenotypic studies, plants were grown on media containing methyl viologen at 0 to 100 nM.

### Phenotypic analyses

Seeds were sown on soil and grown in growth chambers at 22 °C with a 16-h photoperiod (∼130 μmol m^−2^ s^−1^). Relative growth rates were calculated by measuring the dry weight of each sample, each containing 20 seedlings. Tolerance to methyl viologen was assayed by comparing cell damage from leaf discs excised from wild-type and *oxt6* plants. For each ion leakage measurement, three 0.6-cm diameter discs were punched from a fully expanded rosette leaf from plants just prior to bolting. Three leaves were sampled per plant. The discs were placed onto 6 ml distilled water with or without 2.0 μM methyl viologen and sampled after incubation for various times at 22 °C at 80 μmol m^−2^s ^−1^ light (PAR). Dilutions of the incubation fluid were made in water and ion leakage was estimated by measuring the resulting conductivity (Model 61161-362 conductivity meter; VWR International).

To assess the susceptibility of plants to a brief heat shock, stratified seed were sown on MS+sucrose agar plates. Immediately after sowing, control plates were placed at room temperature for 3 hours in the dark, while the experimental plates were placed at 40°C for 3 hours in the dark. Plates were then moved to room temperature and hypocotyl length was measured after 5–7 days of growth in complete darkness.

Low and high temperature growth studies were carried out by growing seedlings on MS+sucrose agar plates for seven days at 22°C before shifting plates to incubators set at different temperatures. After 14-d of additional growth at the designated temperature, seedlings were harvested, dried and weighed. Thirty-six seedlings were weighed for each temperature treatment. All phenotypic assays were repeated at least twice.

### Genetic analysis and mutant complementation

*Oxt6* was backcrossed to wild-type Col and F_2_ seedlings were grown on MS medium containing 100 nM methyl viologen. After 14 days, seedlings with long or short roots were individually transferred to MS medium plates and 2–3 leaves were removed to prepare DNA for PCR analysis. The presence of the T-DNA in segregating individuals was detected by using oligonucleotide primers (Table S6) specific to the neomycin phosphotransferase II (NPTII) gene present within the T-DNA. Genomic DNA flanking the inserted T-DNA was identified by thermal asymmetric interlaced (TAIL) PCR as described by (Liu et al., 1995) using three T-DNA specific primers (Table S6).

A genomic clone encompassing the first two exons of the *OXT6* gene was constructed by PCR amplifying a 3.5-kb fragment containing ∼2- kb upstream of the ATG start codon and 0.5-kb downstream of the translation termination codon present in the 3′-untranslated region of the smaller of the two *OXT6*-derived transcripts (see Results) using appropriate oligonucleotides (OXT6-1 and OXT6-2; Table S6) and wild-type genomic DNA as template. The amplified fragment was ligated into SmaI-digested pBluescript II KS+ and then into the plant transformation vector, pCAMBIA1300, as a BamHI-PstI insert. All constructs were confirmed by sequencing. The plasmids were introduced into *Agrobacterium tumefaciens* GV3101 and *Arabidopsis* plants were transformed as described [49]. Additional details are provided in Methods S1.

### Gene expression analyses

The RNA blotting, RT/PCR, and transcriptional profiling methods are detailed in Methods S1.

### Poly(A) length determination

The protocol described by Preker et al.[50] was followed with slight modifications. Total RNA was isolated from the leaves of soil grown plants using the Trizol reagent. Two μg of RNA were end-labeled with ^32^P-α-3′-dATP using yeast poly(A) polymerase; 10 μl reactions contained 2 μCi of label, a final 3′-dATP concentration of 2 μM, and 500 units of poly(A) polymerase. After 30 minutes, the poly(A) polymerase was inactivated at 90 °C for 3 minutes. Labeled RNAs were then treated with a mixture of RNAse A+RNAse T1 (1 μg and 25 units, respectively, in a 50 μl reaction) in a reaction buffer containing 10 mM Tris-HCl (pH 8.0), 300 mM NaCl, and 50 μg yeast tRNA. After 40 minutes at 37 °C, the reaction was stopped by addition of 1 mg/ml of proteinase K, 5% SDS, 50 mM EDTA, and 200 μg of glycogen and incubated at 42 °C for 30 min. The nuclease resistant poly(A) tails were precipitated in 2.5 M ammonium acetate, 15 mM MgCl_2_, and 2.5 vol of ethanol and pelleted by centrifugation. The recovered nucleic acids were separated on 12% sequencing gels. Size standards consisted of 3′ end -labeled RNAs derived from Decade ^TM^ Marker system (Ambion). When needed, RNAs labeled and treated with RNAses A+T1 were subsequently treated with RNAse H in the presence of oligo-dT; for these reactions, the products of the above reactions were treated in 60 μl with 10 units of RNAse H and 500 ng of oligo-dT_18_.

### 3′-RACE analysis

First-strand cDNA was prepared from 300 ng of total RNA (see the preceding section) in reactions of 10 μl using 60 ng of the 3′-RACE RT primer (Table S6). PCR reactions were then conducted using the nested 3′ primer and the gene-specific primers listed in Table S6. PCR products were purified on agarose gels, cloned into pGEM, and individual clones sequenced using the nested gene specific primer as a sequencing primer. The results presented represent the pooled results of at least two different experiments per gene; all of the sites noted in Figure 7 were seen in all replicates that were performed.

## Supporting Information

Methods S1 Additional Methods.
(0.05 MB DOC)

Table S1 List of Arabidopsis genes whose expression in the oxt6 mutant was at least two-fold different compared with the wild-type. The column designated “wt/mut” is a list of absolute ratios of expression in the wild-type and mutant, respectively; in this column, values greater than two indicate greater expression in the wild-type. The column designated “wt/mut p-value” provides the results of the students t-test for each comparison.
(0.15 MB XLS)

Table S2 List of Arabidopsis genes whose expression in the oxt6 mutant was at least two-fold different compared with the wild-type, and significantly different (p<0.01) when compared with expression in the complemented plants. Groups of genes were extracted from the set of genes that passed the filter and compared with all genes that passed the filter. The results of F-tests, Student t-tests, and z-Tests are presented on the various sheets.
(1.65 MB XLS)

Table S3 The results of sequencing of 3′-RACE clones from nine collections are presented here. The At gene designation is indicated in underlined text; following the AtGID are collections of sequences obtained from wild-type plants, the oxt6 mutant, and the mutant complemented with the smaller At1g30460-encoded RNA. Each line represents a separate sequence. All sequences possessed the poly(A) tract present in the RT primer; this tract has been deleted from the sequences shown here. All sequences read, left to right, 5′→3′, and the 3′-most base denotes the polyadenylation site. Nucleotides other than the poly(A) tract that are that are not templated [48] are denoted in lower case.
(0.06 MB DOC)

Table S4 Statistical analysis of various sets of genes involved in ROS responses. Groups of genes were extracted from the set of genes that passed the filter and compared with all genes that passed the filter.
(0.06 MB XLS)

Table S5 Complete summary of OXT6 expression data in the ATGE developmental series of microarray experiments.
(0.04 MB XLS)

Table S6 Primers and plasmids used in this study.
(0.09 MB DOC)

Table S7 Overlapping probe list for hydrogen peroxide, singlet O2, superoxide and heat shock-induced genes.
(0.11 MB XLS)

## References

[1] Dat J, Vandenabeele S, Vranova E, Van Montagu M, Inze D (2000). Dual action of the active oxygen species during plant stress responses.. Cell Mol Life Sci.

[2] Levine A, Lerner HR (1999). Oxidative stress as a regulator of environmental responses in plants.. Plant responses to environmental stresses: from phytohormones to genome reorganization.

[3] Mittler R, Vanderauwera S, Gollery M, Van Breusegem F (2004). Reactive oxygen gene network of plants.. Trends Plant Sci.

[4] Apel K, Hirt H (2004). Reactive Oxygen Species: Metabolism, oxidative stress, and signal transduction.. Annu Rev Plant Biol.

[5] Asada K, Foyer CH, Mullineaux PM (1994). Production and action of active oxygen species in photosynthetic tissues.. Causes of photooxidative stress and amelioration of defense systems in plants.

[6] Gechev TS, Van Breusegem F, Stone JM, Denev I, Laloi C (2006). Reactive oxygen species as signals that modulate plant stress responses and programmed cell death.. Bioessays.

[7] Foreman J, Demidchik V, Bothwell JH, Mylona P, Miedema H (2003). Reactive oxygen species produced by NADPH oxidase regulate plant cell growth.. Nature.

[8] Gapper C, Dolan L (2006). Control of plant development by reactive oxygen species.. Plant Physiol.

[9] Desikan R, S AH-M, Hancock JT, Neill SJ (2001). Regulation of the Arabidopsis transcriptome by oxidative stress.. Plant Physiol.

[10] Vranova E, Atichartpongkul S, Villarroel R, Van Montagu M, Inze D (2002). Comprehensive analysis of gene expression in Nicotiana tabacum leaves acclimated to oxidative stress.. Proc Natl Acad Sci U S A.

[11] Gadjev I, Vanderauwera S, Gechev TS, Laloi C, Minkov IN (2006). Transcriptomic footprints disclose specificity of reactive oxygen species signaling in Arabidopsis.. Plant Physiol.

[12] Mittler R (2002). Oxidative stress, antioxidants and stress tolerance.. Trends Plant Sci.

[13] Rennenberg H, Polle A (1994). Protection from oxidative stress in transgenic plants.. Biochem Soc Transac.

[14] Rentel MC, Lecourieux D, Ouaked F, Usher SL, Petersen L (2004). OXI1 kinase is necessary for oxidative burst-mediated signalling in Arabidopsis.. Nature.

[15] Anthony RG, Khan S, Costa J, Pais MS, Bogre L (2006). The Arabidopsis protein kinase PTI1-2 is activated by convergent phosphatidic acid and oxidative stress signaling pathways downstream of PDK1 and OXI1.. J Biol Chem.

[16] Davletova S, Schlauch K, Coutu J, Mittler R (2005). The zinc-finger protein Zat12 plays a central role in reactive oxygen and abiotic stress signaling in Arabidopsis.. Plant Physiol.

[17] Rizhsky L, Davletova S, Liang H, Mittler R (2004). The zinc finger protein Zat12 is required for cytosolic ascorbate peroxidase 1 expression during oxidative stress in Arabidopsis.. J Biol Chem.

[18] Davletova S, Rizhsky L, Liang H, Shengqiang Z, Oliver DJ (2005). Cytosolic ascorbate peroxidase 1 is a central component of the reactive oxygen gene network of Arabidopsis.. Plant Cell.

[19] Rossel JB, Wilson PB, Hussain D, Woo NS, Gordon MJ (2007). Systemic and intracellular responses to photooxidative stress in Arabidopsis.. Plant Cell.

[20] Sakamoto H, Maruyama K, Sakuma Y, Meshi T, Iwabuchi M (2004). Arabidopsis Cys2/His2-type zinc-finger proteins function as transcription repressors under drought, cold, and high-salinity stress conditions.. Plant Physiol.

[21] Clayton H, Knight MR, Knight H, McAinsh MR, Hetherington AM (1999). Dissection of the ozone-induced calcium signature.. Plant J.

[22] Evans NH, McAinsh MR, Hetherington AM, Knight MR (2005). ROS perception in Arabidopsis thaliana: the ozone-induced calcium response.. Plant J.

[23] Price AH, Taylor A, Ripley SJ, Griffiths A, Trewavas AJ (1994). Oxidative Signals in Tobacco Increase Cytosolic Calcium.. Plant Cell.

[24] Rentel MC, Knight MR (2004). Oxidative stress-induced calcium signaling in Arabidopsis.. Plant Physiol.

[25] Yang T, Poovaiah BW (2002). Hydrogen peroxide homeostasis: Activation of plant catalase by calcium/calmodulin.. Proc Natl Acad Sci USA.

[26] May M, Leaver CJ (1993). Oxidative stimulation of glutathione synthesis in *Arabidopsis thaliana* suspension cultures.. Plant Physiol.

[27] Griffith OW, Meister A (1979). Potent and specific inhibiton of glutathione synthesis by buthionine sulfoximine (S-*n*-butyl homocysteine sulfoximine).. J Biol Chem.

[28] Delaney KJ, Xu R, Zhang J, Li QQ, Yun KY (2006). Calmodulin interacts with and regulates the RNA-binding activity of an Arabidopsis polyadenylation factor subunit.. Plant Physiol.

[29] Stoilov P, Rafalska I, Stamm S (2002). YTH: a new domain in nuclear proteins.. Trends Biochem Sci.

[30] Buchanan BB, Balmer Y (2005). Redox regulation: a broadening horizon.. Annu Rev Plant Biol.

[31] Gelhaye E, Rouhier N, Navrot N, Jacquot JP (2005). The plant thioredoxin system.. Cell Mol Life Sci.

[32] Busch W, Wunderlich M, Schoffl F (2005). Identification of novel heat shock factor-dependent genes and biochemical pathways in Arabidopsis thaliana.. Plant J.

[33] Addepalli B, Hunt AG (2007). A novel endonuclease activity associated with the Arabidopsis ortholog of the 30-kDa subunit of cleavage and polyadenylation specificity factor.. Nucleic Acids Res.

[34] Forbes KP, Addepalli B, Hunt AG (2006). An Arabidopsis Fip1 homolog interacts with RNA and provides conceptual links with a number of other polyadenylation factor subunits.. J Biol Chem.

[35] Xu R, Ye X, Li Q (2004). AtCPSF73-II gene encoding an Arabidopsis homolog of CPSF 73 kDa subunit is critical for early embryo development.. Gene.

[36] Mittler R, Berkowitz G (2001). Hydrogen peroxide, a messenger with too many roles?. Redox Rep.

[37] Eshdat Y, Holland D, Faltin Z, Ben-Hayyim G (1997). Plant glutathione peroxidases.. Physiol Plant.

[38] Rizhsky L, Liang H, Shuman J, Shulaev V, Davletova S (2004). When defense pathways collide. The response of Arabidopsis to a combination of drought and heat stress.. Plant Physiol.

[39] Shigeoka S, Ishikawa T, Tamoi M, Miyagawa Y, Takeda T (2002). Regulation and function of ascorbate peroxidase isoenzymes.. J Exp Bot.

[40] Gong Z, Dong CH, Lee H, Zhu J, Xiong L (2005). A DEAD box RNA helicase is essential for mRNA export and important for development and stress responses in Arabidopsis.. Plant Cell.

[41] Gong Z, Lee H, Xiong L, Jagendorf A, Stevenson B (2002). RNA helicase-like protein as an early regulator of transcription factors for plant chilling and freezing tolerance.. Proc Natl Acad Sci U S A.

[42] Dong CH, Hu X, Tang W, Zheng X, Kim YS (2006). A putative Arabidopsis nucleoporin, AtNUP160, is critical for RNA export and required for plant tolerance to cold stress.. Mol Cell Biol.

[43] Lee BH, Kapoor A, Zhu J, Zhu JK (2006). STABILIZED1, a stress-upregulated nuclear protein, is required for pre-mRNA splicing, mRNA turnover, and stress tolerance in Arabidopsis.. Plant Cell.

[44] Barabino SM, Ohnacker M, Keller W (2000). Distinct roles of two Yth1p domains in 3′-end cleavage and polyadenylation of yeast pre-mRNAs.. EMBO J.

[45] Tacahashi Y, Helmling S, Moore CL (2003). Functional dissection of the zinc finger and flanking domains of the Yth1 cleavage/polyadenylation factor.. Nucleic Acids Res.

[46] Mandel CR, Kaneko S, Zhang H, Gebauer D, Vethantham V (2006). Polyadenylation factor CPSF-73 is the pre-mRNA 3′-end-processing endonuclease.. Nature.

[47] Ryan K, Calvo O, Manley JL (2004). Evidence that polyadenylation factor CPSF-73 is the mRNA 3′ processing endonuclease.. Rna.

[48] Ok SH, Jeong HJ, Bae JM, Shin JS, Luan S (2005). Novel CIPK1-associated proteins in Arabidopsis contain an evolutionarily conserved C-terminal region that mediates nuclear localization.. Plant Physiol.

[49] Clough SJ, Bent AF (1998). Floral dip: a simplified method for Agrobacterium-mediated transformation of Arabidopsis thaliana.. Plant J.

[50] Preker PJ, Lingner J, Minvielle-Sebastia L, Keller W (1995). The FIP1 gene encodes a component of a yeast pre-mRNA polyadenylation factor that directly interacts with poly(A) polymerase.. Cell.

[51] Jin Y, Bian T (2004). Nontemplated nucleotide addition prior to polyadenylation: a comparison of Arabidopsis cDNA and genomic sequences.. Rna.

