# Supplementary material for: A Polyadenylation Factor Subunit Implicated in Regulating Oxidative Signaling in Arabidopsis thaliana
Source: PLoS One. 2008 Jun 11;3(6):e2410. doi: 10.1371/journal.pone.0002410 (PMC2408970; doi:10.1371/journal.pone.0002410)
Supplement: Methods S1 — Additional Methods. (0.05 MB DOC) [file pone.0002410.s001.doc]

Supporting File 1. Experimental procedures used for determinations of gene expression

*Cloning and expression of At1g30460*

Initially, the shorter transcript cDNA (AtCPSF30) was cloned based on the predicted amino acid homology to bovine CPSF30 and yeast Yth1p, using cloning and RACE protocols as described (Xu et al., 2004). The primers used for cloning were OXT6-3 and OXT6-4 (Table S6). The larger cDNA was identified by 5′ and 3′ RACE, and cloned using the primers OXT6-5 and OXT6-6 (Table S6). The sequences for these clones have been deposited in Genbank (accessions AY140901 and EU250988), for the short (AtCPSF30) and long (AtCPSF30*-YT521B) polypeptides, respectively.

RNA blotting was performed with total RNA isolated from soil-grown wild-type and *oxt6* plants. RNA was isolated using the Trizol Reagent (Life Technologies, Rockville, MD) was separated on 1% agarose gels in the presence of 0.5M formaldehyde, and the separated RNA transferred to membranes as described (Wang et al., 2004). Filters were hybridized with labeled probes prepared from the parts of the *OXT6* gene shown in Figure 4A; these probes were generated by PCR, using primers (listed in Table S6) OXT6-3 and OXT6-7 for the short transcript (probe 1 in Figure 4A), and primers OXT6-9 and OXT6-10 for the probe targeted to the downstream portion of the gene (probe 2 in Figure 4A).

RT-PCR determinations of *OXT6* gene expression in different tissues was conducted as described previously (Wang et al., 2004). For RT-PCR determinations following MV treatment, 14-d-old seedlings grown on MS plus 1% (w/v) sucrose medium were carefully removed from the agar medium and transferred to three layers of filter paper (3MM, Whatman) soaked with MS liquid medium containing 2 mM methyl viologen or MS medium alone. After 0, 1, 10, or 24 hours, total RNA was extracted from leaves using the TRIzol Reagent (Invitrogen). DNA was removed from the RNA preparations with DNase I (Amp Grade, Invitrogen). One microgram total RNA was reverse transcribed with decamer oligonucleotides included in the RETROscript kit (Ambion) following the supplier's instructions. PCR reactions were performed using the primers OXT6-3 and OXT6-7 for the short, CPSF30 transcript, and OXT6-3 and OXT6-10 for the long CPSF30-YT521-B transcript for analysis in Figure 4D. RT-PCR products were separated on 1.5 or 2.5% (w/v) agarose gels, stained with SYBR Green I (Molecular Probes), and band intensities were quantified with an image analyzer (Model FLA-2000, Fujifilm, Japan). The Arabidopsis actin 2 (*ACT2*) gene (An et al., 1996) was used as a control in this experiment, using the gene-specific primers described previously (Panchuk et al., 2002).

To conduct the *in silico* gene analysis of *OXT6* gene expression (Figure 4C), expression data for the *OXT6* gene (At1g30460, probe 261798_at) was downloaded from the NASC web site (<http://affymetrix.arabidopsis.info/narrays/help/psp-wbubn.html>). Normalized expression values were extracted, compiled (Supplemental Table S4), and analyzed as indicated in the text and figure legends. The sample key for the experiments used here is presented in Supplemental Table S4; this key connects the individual experiments with the various plant sample types and experimental variables.

*Transcriptional profiling and analysis*

*Arabidopsis* plants (the wild-type, *oxt6* mutant, and two independent *oxt6* transformants each carrying a gene encoding just the smaller of the two At1g30460-encoded proteins) were seeded and grown in soil at 22-26°C in an 8 hr light/16 hr dark regime (under Phillips F40CW 34W fluorescent lights, with an intensity of 55 µmol m^-2^ sec^-1^). Plants were harvested for RNA isolation after 30 days; for this, six samples derived from 10-15 plants were prepared for each line. RNA was isolated using the Invitrogen Trizol Reagent and further purified with the Qiagen RNeasy Plant Mini Kit, and tested for quality by gel electrophoresis and inspection of the stable RNA bands. Preparations with the highest quality were selected for labeling and hybridization to chips. Ten samples were selected for study – three wild-type, three *oxt6*, and two from each of the complemented lines. Analysis of independent complemented plants minimizes the possible effects that may arise due to T-DNA insertion. RNA labeling, hybridization to Affymetrix *Arabidopsis* gene chips (ATH1 Genome Array), and initial analysis of the chips was performed by the University of Kentucky Microarray Core Facility (<http://www.mc.uky.edu/UKMicroArray/>). CEL files were further analyzed using the ArrayAssist software package (Stratagene) with the PLIER normalization protocol. Correlation coefficients for comparisons of expression data for chips within an experimental group (e.g., pairwise comparison of all expression data for each of the three wild-type chips) were no lower than 0.95 (not shown), indicating that the chip-to-chip variability was minimal in this study. Data for this experiment have been deposited in the GEO repository (accession GSE7211).

Gene lists were derived from pairwise comparisons. Briefly, probes called “absent” by the PLIER analysis on any of the three (for the wild-type and *oxt6* mutant) or four (for the complemented lines) chips in the analysis were removed from the analysis stream. For each probe, the expression ratios for the respective comparisons were then calculated and significance levels (p values from Student’s t-tests) were compiled. The results revealed 850 probes whose expression was at least two-fold higher or lower, at a confidence level P < 0.01, in the *oxt6* mutant than in the wild-type (Supplemental Table S6). To narrow the list of 850 probes down to those responsive to just the smaller *OXT6*-encoded mRNA, they were compared with the set of probes that were significantly different between the *oxt6* mutant and the two complemented plant lines described in the Results. This exercise yielded 398 probes that were also significantly different, at the P<0.01 level, in the mutant compared with the complemented lines (Table S7). Because the stress-tolerant phenotypes of the wild-type and complemented lines are similar, those probes in Table S7 that were less than two-fold different in the wild-type/complemented comparison were retained for final compilation. In addition, probes that had opposing trends in the *oxt6* mutant and the complemented lines (abundance in the mutant less than in the wild-type, and abundance in the complemented plants greater than in the wild-type, and the converse of these cases) were also retained. This exercise yielded 352 probes whose expression correlated with the stress-tolerant phenotype. Because of some ambiguity in the probe-gene correspondence, this set of 352 probes identified 362 genes (Table S1).

In some instances, RNA blot analysis was performed to corroborate the microarray results. These studies were conducted as described elsewhere (Addepalli et al., 2004), using probes derived from the plasmids listed in Table S6. Template for labeled probe for one of the genes mentioned in Table 1 (for At5g06690) was produced by RT/PCR as described (Addepalli et al., 2004), using *Arabidopsis* total RNA and the primers indicated in Table S6. The results of this corroboration are available upon request.

References:

**Addepalli, B., Meeks, L.R., Forbes, K.P., and Hunt, A.G.** (2004). Novel alternative splicing of mRNAs encoding poly(A) polymerases in *Arabidopsis*. Biochim Biophys Acta **1679,** 117-128.

**An, Y.Q., McDowell, J.M., Huang, S., McKinney, E.C., Chambliss, S., and Meagher, R.B.** (1996). Strong, constitutive expression of the *Arabidopsis* ACT2/ACT8 actin subclass in vegetative tissues. Plant J **10,** 107-121.

**Panchuk, I.I., Volkov, R.A., and Schoffl, F.** (2002). Heat stress- and heat shock transcription factor-dependent expression and activity of ascorbate peroxidase in *Arabidopsis*. Plant Physiol **129,** 838-853.

**Wang, H., Caruso, L.V., Downie, A.B., and Perry, S.E.** (2004). The embryo MADS domain protein AGAMOUS-Like 15 directly regulates expression of a gene encoding an enzyme involved in gibberellin metabolism. Plant Cell **16,** 1206-1219.

**Xu, R., Ye, X. and Li, Q.Q.** (2004). AtCPSF73-II gene encoding an *Arabidopsis* homolog of CPSF 73 kDa subunit is critical for early embryo development. Gene **324**, 35-45.
