# Supplementary material for: A Polyadenylation Factor Subunit Implicated in Regulating Oxidative Signaling in Arabidopsis thaliana
Source: PLoS One. 2008 Jun 11;3(6):e2410. doi: 10.1371/journal.pone.0002410 (PMC2408970; doi:10.1371/journal.pone.0002410)
Supplement: Table S3 — The results of sequencing of 3′-RACE clones from nine collections are presented here. The At gene designation is indicated in underlined text; following the AtGID are collections of sequences obtained from wild-type plants, the oxt6 mutant, and the mutant complemented with the smaller At1g30460-encoded RNA. Each line represents a separate sequence. All sequences possessed the poly(A) tract present in the RT primer; this tract has been deleted from the sequences shown here. All sequences read, left to right, 5′→3′, and the 3′-most base denotes the polyadenylation site. Nucleotides other than the poly(A) tract that are that are not templated [48] are denoted in lower case. (0.06 MB DOC) [file pone.0002410.s004.doc]

**Supporting Table S3 – compilation of 3′-RACE sequences.**

**UBC28 – At1g64230**

wild-type sequences:

CCATGAAATAAGTTAGATTCCTATGTTTTATCATCTCTTTGTTTGAAACCTCTTTAATCTCAAACAAAAACATTACTcc

CCATGAAATAAGTTAGATTCCTATGTTTTATCATCTCTTTGTTTGAAACCTCTTTAATCTCAAACAAAAACATTACTTCACC

CCATGAAATAAGTTAGATTCCTATGTTTTATCATCTCTTTGTTTGAAACCTCTTTAATCTCAAACAAAAACATTACTTCACCTCTTTATTATCCATATGTTACCCTATCTTTGTTTCTATGCTTTTTCCAAAAAATTATAAGAAAAATTATTTATG

CCATGAAATAAGTTAGATTCCTATGTTTTATCATCTCTTTGTTTGAAACCTCTTTAATCTCAAACAAAAACATTACTTCACCTCTTTATTATCCATATGTTACCCTATCTTTGTTTCTATGCTTTTTCCAAAAAATTATAAGAAAAATTATTTATG

CCATGAAATAAGTTAGATTCCTATGTTTTATCATCTCTTTGTTTGAAACCTCTTTAATCTCAAACAAAAACATTACTTC

CCATGAAATAAGTTAGATTCCTATGTTTTATCATCTCTTTGTTTGAAACCTCTTTAATCTCAAACAAAAACATTACTcc

CCATGAAATAAGTTAGATTCCTATGTTTTATCATCTCTTTGTTTGAAACCTCTTTAATCTCAAACAAAGACATTACTTCACCcc

CCATGAAATAAGTTAGATTCCTATGTTTTATCATCTCTTTGTTTGAAACCTCTTTAATCTCAAACAAAAACATTACTcc

CCATGAAATAAGTTAGATTCCTATGTTTTATCATCTCTTTGTTTGAAACCTCTTTAATCTCAAACAAAAACATTACTTC

CCATGAAATAAGTTAGATTCCTATGTTTTATCATCTCTTTGTTTGAAACCTCTTTAATCTCAAACAGAAACATTACTTCACCTCTTTATTATCCATATGTTACCCTATCTTTGTTTCTATGCTTTTTCCAAAAAATTATAAGAAAAATTATTTATGT

*oxt6* sequences:

CCACGAAATAAGTTAGATTCCTATGTTTTATCATCT

CCACGAAATAAGTTAGATTCCTATGTTTTATCATCTCTTTGTTT

CCACGAAATAAGTTAGATTCCTATGTTTTATCATCT

CCATGAAATAAGTTAGATTCCTATGTTTTATCATCTCTTTGTTTGAAACCTCTTTAATCTCAAACAAGAACATTACTTCACCCCTTTATTATCCAc

CCATGAAATAAGTTAGATTCCTATGTTTTATCATCTCTTTGTTTGAAACCTCTTTAATCTCAAACAAAAACATTACTTCACCTCTTTATTATCCATATGTTCCCTATCTTTGTTTCTATGCTTTTTCC

CCATGAAATAAGTTAGATTCCTATGTTTTATCATCTCTTTGTTTGAAACCTCTTTAATCTCAAACAAAAACATTACTTCACCTCTTTATTATCCATATGTTCCCTATCTTTGTTTCTATGCTTTTTCC

CCATGAAATAAGTTAGATTCCTATGTTTTATCATCTCTTTGTTTGAAACCTCTTTAATCTCAAACAAGAACATTACTTCACCCCTTTATTATCCAc

CCATGAAATAAGTTAGATTCCTATGTTTTATCATCTCTTTGTTTGAAACCTCTTTAATCTCAAACAAAAACATTACTTCACCTCTTTATTATCCAc

CCATGAAATAAGTTAGATTCCTATGTTTTATCATCTCTTTGTTTGAAACCTCTTTAATCTC

CCATGAAATAAGTTAGATTCCTATGTTTTATCATCTCTTTGTTTGAAACCTCTTTAATCTCAAACAAAAACATTACTTCACCTCTTTATTATCCATATGTTCCCTATCTTTGTTTCTATGCTTTTTCC

CCATGAAATAAGTTAGATTCCTATGTTTTATCATCTCTTTGTTTGAAACCTCTTTAATCTCAAACAAGAACATTACTTCACCCCTTTATTATCCAc

complement sequences:

CCATGAAATAAGTTAGATTCCTATGTTTTATCATCTCTTTGTTTGAAACCTCTTTAATCTCAAACAAAAACATTACTcc

CCATGAAATAAGTTAGATTCCTATGTTTTATCATCTCTTTGTTTGAAACCTCTTTAATCTCAAACAAAAACATTACTTCACCTC

CCATGAAATAAGTTAGATTCCTATGTTTTATCATCTCTTTGTTTGAAACCTCTTTAATCTCAAACAAAAACATTACTTCACCTCTTTA

CCATGAAATAAGCTAGATTCCTATGTTTTATCATCTCTTTGTTTGAAACCTCTTTAATCTCAAGCAAAAACATTACTTCACCT

CCATGAAATAAGTTAGATTCCTATGCTTTATCATCTCTTTGTTTGAAACCTCTTTAATCTCAAACAAAAACATTACTTCACCc

CCATGAAATAAGTTAGATTCCTATGTTTTATCATCTCTTTGTTTGAAACCTCTTTAATcTCAAACAAAAACaTTACTTCCCCTCTTT

CCATGAAATAAGTTAGATTCCTATGTTTTATCATCTCTTTGTTTGAAACCTCTTTAATCTCAAACAAAAACATTACTTCACCTCTTTAT

CCATGAAATAAGTTAGATTCCTATGTTTTATCATCTCTTTGTTTGAAACCTCTTTAATCTCAAACAAAAACACTACTTC

CCATGAAATAAGTTAGATTCCTATGTTTTATCATCTCTTTGTTTGGAACCTCTTTAATCTCAAACAAAAACATTACTTC

CCATGAAATAAGTTAGATTCCTATGTTTTATCATCTCTTTGTTTGAAACCTCTTTAATCTCAAACAAAAACATTACTTCACCTCTTTATTATCCATATGTTCCCTATCTTTGTTTC

CCATGAAATAAGTTAGATTCCTATGTTTTATCATCTCTTTGTTTGAAACCTCTTTAATCTCAAACAAAAACATTACTTCACCTCTTTATTATCCATATGTTCCCT

CCATGAAATAAGTTAGATTCCTATGTTTTATCATCTCTTTGTTTGAAACC

**Metallothionein -At3g09390**

wild-type sequences:

TCTGCCATGTGATGAGTTTGTACTTCCAGTGGAATGATAATAATATTATAGTTTTAAATC

TCTGCCATGTGATGAGTTTGTACTTCCAGTGGAATGATAATAATATTATAGTTTTAAATC

TCTGCCATGTGATGAGTTTGTACTTCCAGTGGAATGATAATAATATTATAGTTTTAAATC

TCTGCCATGTGATGAGTTTGTACTTCCAGTGGAATGATAATAATATTATAGTTTT

TCTGCCATGTGATGNGTTTGTACTTCCAGTGGAATGATAATAATATTATAGTTTT

TCTGCCATGTGATGAGTTTGTACTTCCAGTGGNATGATAATAATATTATAGTTTT

TCTGCCATGTGATGAGTTTGTACTTCCTAGTGGAATGATAATAATATTATAGTTTTAAATCT

TCTGCCATGTGATGAGTTTGTACTTCCAGTGGAATGATAATAATATTATAGTTTT

TCTGCCATGTGATGAGTTTGTACTTCCAGTGGAATGATAATAATATTATAGTTTTAAATCT

TCTGCCATGTGATGAGTTTGTACTTCCAGTGGNATGATAATAATATTATAGTTTT

TCTGCCATGTGATGAGTTTGTACTTCCAGTGGNATGATAATAATATTATAGTTTT

*oxt6* sequences:

TCTGCCATGTGATGAGTTTGTACTTCCAGTGGAATGATAATAATATTATAGTT

TCTGCCATGTGATGAGTTTGTACTTCCAGTGGAATGATAATAATATTATAGTT

TCTCCCATGTGATGAGTTTGTACTTCCAGTGGAATGATAATAATATTATAGTTT

TCTGCCATGTGATGAGTTTGTACTTCCAGTGGAATGATAATAATATTATAGTTTTAAATCTCTTTGTTCGTTGGGTTTTCTTCATTGG

TCTGCCATGTGATGAGTTTGTACTTCCAGTGGAATGATAATAATATTATAGTTTT

TCTGCCATGTGATGAGTTTGTACTTCCAGTGGAATGATAATAATATTATAGTTTT

TCTGCCATGTGATGAGTTTGTACTTCCAGTGGAATGATAATAATATTAT

TCTGCCATGTGATGAGTTTGTACTTCCAGTGGAATGATAATAATATTATAGTTTT

TCTGCCATGTGATGAGTTTGTACTTCCAGTGGAATGATAATAATATTATAGTTTT

TCTGCCATGTGATGAGTTTGTACTTCCAGTGGAATGATAATAATATTATAGTTTTAAATCTCTTT

complement sequences:

TCTGCCATGTGATGAGTTTGTACTTCCAGTGGAATGATAATAATATTATAGTTTTAAATCTCTTTGTTCGTTGGGTTTTCTTC

TCTGCCATGTGATGAGTTTGTACTTCCAGTGGAATGATAATAATATTATAGTTTTAAATCTCTTTGTTCGTTGGGTTTTCTTC

TCTGCCATGTGATGAGTTTGTACTTCCAGTGGAATGATAATAATATTATAGTTTAAAATCTCTTTGTTCGTTGGGTTTTCTTCATTGGTCCTCCATTGTTTCTAAATTTTGAAGAAGT

TCTGCCATGTGATGAGTTTGTACTTCCAGTGGAATGATAATAATATTATAGTTTAAAATCTCTTTGTTCGTTGGGTTTTCTTCATTGGTCCTCCATTGTTTCTAAATTTTGAAGAA

TCTGCCATGTGATGAGTTTGTACTTCCAGTGGAATGATAATAATATTATAGTTTAAAATCTCTTTGTTCGTTGGGTTTTCTTCATTGGTCCTCCATTGTTTCTAAATTTTGAAGAA

TCTGCCATGTGATGAGTTTGTACTTCCAGTGGAATGATAATAATATTATAGTTTTAAATCCCTTTGTTCGTTGGGTTTTCTTC

TCTGCCATGTGATGAGTTTGTACTTCCAGTGGAATGATAATAATATTATAGTTTTAAATC

TCTGCCATGTGATGAGTTTGTACTTCCAGTGGAATGATAATAATATTACAGTTTT

TCTGCCATGTGATGAGTTTGTACTTCCAGTGGAATGATAATAATATTACAGTTTT

TCTGCCATGTGATGAGTTTGTACTTCCAGTGGAATGATAATAATATTATAGTTTT

TCTGCCATGTGATGAGTTTGTACTTCCAGTGGAATGATAATAATATTACAGTTTT

TCTGCCATGTGATGAGTTTGTACTTCCAGTGGAATGATAATAATATTATAGTTTT

**Thionin - At5g36910**

wild-type sequences:

GGGTTTTTGATCCTAGATTGTATTATATCCCTTCTACTTGTCTTTCTCTAAATAAATAATAATGTGTCCTTCTCGACT

GGGTTTTTGATCCTAGATTGTATTATATCCCTTCTACTTGTCTTTCTCTAAATAAATAATAATGTGTCCTTCTCGACT

GGGTTTTTGATCCTAGATTGTATTATATCCCTTCTACTTGTCTTTCTCTAAATAAATAATGATGTGTCCTTCTCGACT

AGGTTTTTGATCCTAAAATGGATTATATCCCTTCTACTTGTCTTTCTCTAAATAAAT

GGGTTTTTTATCCTAAAATGGATTATATCCCTTCTACTTGCCTTTCTCTAAATAAAT

GGGTTTTTGATCCTAGATTGTATTATATCCCTTCTACTTGTCTTTCTCTAAATAAATAATAATGTGTCCTTCTCGAC

GGGTTTTTGATCCTAGATTGTATTATATCCCTTCTACTTGTCTTTCTCTAAATAAATAATAATGTGTCCTTCTCGACT

GGGTTTTTGATCCTAGATTGTATTATATCCCTTCTACTTGTCTTTCTCTAAATAAATAATAATGTGTCCTTCTCGAC

GGGTTTTTGATCCTAGATTGTATTATATCCCTTCTACTTGTCTTTCTCTAAATAAATAATGATGTGTCCTTCTCGACT

GGGTTTTTGATCCTAGATTGTATTATATCCCTTCTACTTGTCTTTCTCTAAATAAATAATAATGTGTCCTTCTCGACT

GGGTTTTTGATCCTAGATTGTATTATATCCCTTCTACTTGTCTTTCTCTAAATAAATAATAATGTGTCCTTCTCGACT

GGGTTTTTGATCCTAGATTGTATTATATCCCTTCTACTTGTCTTTCTCTAAATAAATAATAATGTGTCCTTCTCGACT

GGGTTTTTGATCCTAGATTGTATTATATCCCTTCTACTTGTCTTTCTCTAAATAAATAATAATGTGTCCTTCTCGACT

GGGTTTTTGATCCTAGATTGTATTATATCCCTTCTACTTGTCTTTCTCTAAATAAATAATAATGTGTCCTTCTCGACT

GGGTTTTTGATCCTAGATTGTATTATATCCCTTCTACTTGTCTTTCTCTAAATAAATAATAATGTGTCCTTCTCGAC

*oxt6* sequences:

GGGTTTTTGATCCTAGATTGTATTATATCCCTTCTACGTTGTCTTTCTc

GGGTTTTTGATCCTAGATTGTATTATATCCCTTCTACTTGTCTTTCTCgat

GGGTTTTTGATCCTAGATTGTATTATATCCC

GGGTTTTTGATCCTAGATTGTATTATATCCCTTCTATTTGTCTTTCTCTAAAT

GGGTTTTTGATCCTAGATTGTATTATATCCCTTCTATTTGTCTTTCTCTAAAT

GGGTTTTTGATCCTAGATTGTATTATATCCCTTCTACTTGTCTTTCTCgat

GGGTTTTTGATCCTAGATTGTATTATATCCCTTCTACTTGTCTTTCTCgat

GGGTTTTTGATCCTAGATTGTATTATATCCCTTCTACTTGTCTTTCTCgat

GGGTTTTTGATCCTAGATTGTATTATATCCCTTCTACTTGTCTTTCTCgat

GGGTTTTTGATCCTAGATTGTATTATATCCCTTCTACTTGTCTTTCTCgat

GGGTTTTTGATCCTAGATTGTATTATATCCCTTCTACTTGTCTTTCTCgat

GGGTTTTTGATCCTAGATTGAATTATATCCCTT

GGGTTTTTGATCCTAGATTGTATTATATCCCTTCTACTTGTCTTTCTCgat

GGGTTTTTGATCCTAGATTGTATTATATCCCTTCTACTTGTCTTTCTCgat

GGGTTTTTGATCCTAGATTGTATTATATCCCTTCTACTTGTCTTTCTCgat

GGGTTTTTGATCCTAGATTGTATTATATCCCTTCTACTTGTCCTTCTCT

complement sequences:

GGGATTTTGATCCTAGATTGTATTATATCCCTTCTACCTGTCTTTCTCT

GGGTTTTTGATCCTAGATTGTATTATATCCCTTCTACTTGTCTTTCTCTAg

GGGTTTTTGATCCTAGATTGTATTATATCCCTTCTACTTGTCTTTCTCTAAAT

GGGTTTTTGATCCTAGATTGTATTATATCCCT

GGGTTTTTGATCCTAGATTGTATTATATCCCTTCTACTTGTCTTTCTCT

GGGTTTTTGATCCTAGATTGTATTATATCCCTTCTACTTGTCTTTCTCTAAATAAATAATAATGTGTCCTTCTCGACT

GGGTTTTTGATCCCAGATTGTATTATATCCCTTCTACTTGTCTTTCTCTAAATAAAT

GGGTTTTTGATCCCAGATTGTATTATATCCCTTCTACTTGTCTTTCTCTAAATAAAT

GGGTTTTTGATCCTAGATTGTATTATATCCCTTCTACTTGTCTTTCTCTAg

GGGTTTTTGATCCTAGATTGTATTATATCCCTTCTACTTGTCTTTCTCTAAAT

GGGTTTTTGATCCTAGATTGTATTATATCCCTTCTACTTGTCTTTCTCTAAAT

GGGTTTTTGATCCTAGATTGTATTATATCCCTTCTACTTGTCTTTCTCTAg

GGGTTTTTGATCCTAGATTGTATTATATCCCTTCTACTTGTCTTTCTCTAg

GGGTTTTTGATCCTAGATTGTATTATATCCCTTCTACTTGTCTTTCTCTAg

GGGTTTTTGATCCTAGGTCGTATTATATCCCTTCTACTTGTCTCTCTCTAg

GGGTTTTTGATCCTAGGTCGTATTATATCCCTTCTACTTGTCTCTCTCTAg

GGGTTTTTGATCCTAGGTCGTATTATATCCCTTCTACTTGTCTCTCTCTAg

GGGTTTTTTATCCTAGATTGTATTATATCCCTTCTACTTGTTTTTCTCT

**rbcS – At5g38410**

wild-type sequences:

TTCTTTTTCTCTTTATGAGACAATTTCTATCGGATTGTCAAATGTCTGATTTATGAATATGTAATTTAT

TTCTTTTTCTCTTTATGAGACAATTTCTATCGGATTGTCAAATGTCTGATTTATGAATATGTAATTTATAT

TTCTTTTTCTCTTTATGAGACAATTTCTATCGGATTGTCAAATGTCTGATTTATGAATATGTAATTTATATATCCGTGCGTCTTGATT

TTCTTTTTCTCTTTATGAGACAATTTCTATCGGATTGTCAAATGTCTGATTTATGAATATGTAATTTATAT

TTCTTTTTCTCTTTATGAGACAATTTCTATCGGATTGTCAAATGTCTGATTTATGAATATGTAATTTATAT

TTCTTTTTCTCTTTATGAGACAATTTCTATCGGATTGTC

TTCTTTTTCTCTTTATGAGACAATTTCTATCGGATTGTCAAATGTCTGATTTATGAATATGTAATTTATATATCCGTGCGTCTTGATT

TTCTTTTTCTCTTTATGAGACAATTTCTATCGGATTGTC

TTCTTTTTCTCTTTATGAGACAATTTCTATCGGATTGTCAAATGTCTGATTTATGAATATGTAATTTAT

TTCTTTTTCTCTTTATGAGACAATTTCTATCGGATTGTCAAATGTCTGATTTATGAATATGTAATTTATAT

TTCTTTTTCTCTTTATGAGGCAATTTCTATCGGATTGTCAAATGTCTGATTTATGAATATGTAATTTATAT

TTCTTTTTCTCTTTATGAGACAATTTCTATCGGATTGTCAAATGTCTGATTTATGAATATGTAATTTATATATCCGTGCGTCTTGATTTTTTCCGATGGTTAACTAGTTTGAAAATTTCCGATGAGATAAGACAACATACAAAAAATCGAATAAATTGTGT

TTCTTTTTCTCTTTATGAGACAATTTCTATCGGATTGTCAAATGTCTGATTTATGAATATGTAATTTAT

TTCTTTTTCTCTTTATGAGACAATTTCTATCGGATTGTCAAATGTCTGATTTATGAATATGTAATTTATAT

TTCTTTTTCTCTTTATGAGACAATTTCTATCGGATTGTCAAATGTCTGATTTATGAATATGTAATTTATATATCCGTGCGTCTTGATTTTTTCCGATGGTT

TTCTTTTTCTCTTTATGAGACAATTTCTATCGGATTGTCAAATGTCTGATTTATGAATATGTAATTTATAT

TTCTTTTTCTCTTTATGAGACAATTTCTATCGGATTGTCAAATGTCTGATTTATGAATATGTAATTTATAT

TTCTTTTTCTCTTTATGAGACAATTTCTATCGGATTGTCAAATGTCTGATTTATGAATATGTAATTTATAT

oxt6 sequences:

TTCTTTTTCTCTTTATGAGACAATTTCTATCGGATTGTCAAATGTCTGATTTATGAATATGTAATTTATATATCCGTGCGTCTTGATTTTTTCCGATGGTTAACTAGTTTG

TTCTTTTTCTCTTTATGAGACAATTTCTATCGGATTGTCAAATGTCTGATTTATGAATATGTAATTTAT

TTCTTTTTCTCTTTATGAGACAATTTCTATCGGATTGTCAAATGTCTGATTTATGAATATGTAATTTATAT

TTCTTTTTCTCTTTATGAGACAATTTCTATCGGATTGTCAAATGTCTGATTTATGAATATGTAATTTATAT

TTCTTTTTCTCTTTATGAGACAATTTCTATCGGATTGTCAAATGTCTGATTTATGAATATGTAATTTAT

TTCTTTTTCTCTTTATGAGACAATTTCTATCGGATTGTCAAATGTCTGATTTATGAATATGTAATTTATATATCCGTGCGTCTTGATTTTTTCCGATGGTTAACTAGTTTG

TTCTTTTTCTCTTTATGAGACAATTTCTATCGGATTGTCAAATGTCTGATTTATGAATATGTAATTTATATATCCGTGCGTCTTGATTTTTTCCGATGGTTAACTAGTTTG

TTCTTTTTCTCTTTATGAGACAATTTCTATCGGATTGTCAAATGTCTGATTTATGAATATGTAATTTATAT

TTCTTTTTCTCTTTATGAGACAATTTCTATCGGATTGTCAAATGTCTGATTTATGAAT

TTCTTTTTCTCTTTATGAGACAATTTCTATCGGATTGTCAAATGTCTGATTTATGAATATGTAATTTATATATCCGTGCGTCTTGATTTTTTCCGATGGTTAACT

TTCTTTTTCTCTTTATGAGACAATTTCTATCGGATTGTCAAATGTCTGATTTATGAATATGTAATTTAT

TTCTTTTTCTCTTTATGAGACAATTTCTATCGGATTGTCAAATGTCTGATTTATG

TTCTTTTTCTCTTTATGAGACAATTTCTATCGGATTGTCAAATGTCTGATTTATGAATATGTAATTTATATATCCGTGCGTCTTGATTTTTTCCGATGGTTAACTAGTTTG

TTCTTTTTCTCTTTATGAGACAATTTCTATCGGATTGTCAAATGTCTGATTTATGAATATGTAATTTATAT

TTCTTTTTCTCTTTATGAGACAATTTCTATCGGATTGTCAAATGTCTGATTTATGAATATGTAATTTAT

TTCTTTTTCTCTTTATGAGACAATTTCTATCGGATTGTCAAATGTCTGATTTATGAATATGTAATTTAT

TTCTTTTTCTCTTTATGAGACAATTTCTATCGGATTGTCAAATGTCTGATTTATGAATATGTAATTTATATATCCGTGCGTCTTGATTTTTTCCGATGGTT

TTCTTTTTCTCTTTATGAGACAATTTCTATCGGATTGTCAAATGTCTGATTTATGAATATGTAATTTATAT

TTCTTTTTCTCTTTATGAGACAATTTCTATCGGATTGTCAAATGTCTGATTTATGAATATGTAATTTAT

TTCTTTTTCTCTTTATGAGACAATTTCTATCGGATTGTCAAATGTCTGATTTATGAATATGTAATTTAT

TTCTTTTTCTCTTTATGAGACAATTTCTATCGGATTGTCAAATGTCTGATTTATGAATATGTAATTTATATATCCGTGCGTCTTGATTTTTTCCGATGGTT

TTCTTTTTCTCTTTATGAGACAATTTCTATCGGATTGTCAAATGTCTGATTTATGAATATGTAATTTAT

TTCTTTTTCTCTTTATGAGACAATTTCTATCGGATTGTCAAATGTCTGATTTATGAATATGTAATTTAT

TTCTTTTTCTCTTTATGAGACAATTTCTATCGGATTGTCAAATGTCTGATTTATGAATATGTAATTTATAT

TTCTTTTTCTCTTTATGAGACAATTTCTATCGGATTGTCAAATGTCTGATTTATGAATATGTAATTTATATATCCGTGCGTCTTGATTTTTTCCGATGGTTAACT

TTCTTTTTCTCTTTATGAGACAATTTCTATCGGATTGTCAAATGTCTGATTTATGAATATGTAATTTATATATCCGTGCGTCTTGATTTTTTCCGATGGTTAACT

complement sequences:

TTCTTTTTCTCTTTATGAGACAATTTCTATCGGATTGTCAAATGTCTGATTTATGAATATGTAATTTATAT

TTCTTTTTCTCTTTATGAGACAATTTCTATCGGATTGTCAAATGTCTGATTTATGAATATGTAATTTATAT

TTCTTTTTCTCTTTATGAGACAATTTCTATCGGATTGTCAAATGTCTGATTTATGAATATGTAATTTATAT

TTCTTTTTCTCTTTATGAGACAATTTCTATCGGATTGTCAAATGTCTGATTTATGAATATGTAATTTATAT

TTCTTTTTCTCTTTATGAGACAATTTCTATCGGATTGTCAAATGTCTGATTTATGAATATGTAATTTATAT

TTCTTTTTCTCTTTATGAGACAATTTCTATCGGATTGTCAAATGTCTGATTTATGAATATGTAATTTATAT

TTCTTTTTCTCTTTATGAGACAATTTCTATCGGATTGTCAAATGTCTGATTTATGAATATGTAATTTATAT

TTCTTTTTCTCTTTATGAGACAATTTCTATCGGATTGTCAAATGTCTGATTTATGAATATGTAATTTATAT

TTCTTTTTCTCTTTATGAGACAATTTCTATCGGATTGTCAAATGTCTGATTTATGAATATGTAATTTATAT

TTCTTTTTCTCTTTATGAGACAATTTCTATCGGATTGTCAAATGTCTGATTTATGAATATGTAATTTATAT

TTCTTTTTCTCTTTATGAGACAATTTCTATCGGATTGTCAAATGTCTGATTTATGAATATGTAATTTAT

TTCTTTTTCTCTTTATGAGACAATTTCTATCGGATTGTCAAATGTCTGATTTATGAATATGTAATTTATAT

TTCTTTTTCTCTTTATGAGACAATTTCTATCGGATTGTCAAATGTCTGATTTATGAATATGTAATTTATAT

TTCTTTTTCTCTTTATGAGACAATTTCTATCGGATTGTCAAATGTCTGATTTATGAATATGTAATTTAT

TTCTTTTTCTCTTTATGAGACAATTTCTATCGGATTGTCAAATGTCTGATTTATGAATATGTAATTTATAT

TTCTTTTTCTCTTTATGAGACAATTTCTATCGGATTGTCAAATGTCTGATTTATGAATATGTAATTTATAT

TTCTTTTTCTCTTTATGAGACAATTTCTATCGGATTGTCAAATGTCTGATTTATGAATATGTAATTTAT

TTCTTTTTCTCTTTATGAGACAATTTCTATCGGATTGTCAAATGTCTGATTTATGAATATGTAATTTATAT

TTCTTTTTCTCTTTATGAGACAATTTCTATCGGATTGTCAAATGTCTGATTTATGAATATGTAATTTATAT

TTCTTTTTCTCTTTATGAGACAATTTCTATCGGATTGTCAAATGTCTGATTTATGAATATGTAATTTAT
