# Supplementary material for: A Polyadenylation Factor Subunit Implicated in Regulating Oxidative Signaling in Arabidopsis thaliana
Source: PLoS One. 2008 Jun 11;3(6):e2410. doi: 10.1371/journal.pone.0002410 (PMC2408970; doi:10.1371/journal.pone.0002410)
Supplement: Table S6 — Primers and plasmids used in this study. (0.09 MB DOC) [file pone.0002410.s007.doc]

Supporting Table S6. Oligonucleotides and plasmids used in this research

| Designation | source (AtGID) | sequence (5′->3′) or plasmid designation | used for |
| --- | --- | --- | --- |
| primers |  | sequence (5′->3′) |  |
|  |  |  |  |
| NPT-5′ | NPTII gene | CCTGTCCGGTGCCCTGAATG | PCR, confirming T-DNA insert |
| NPT-3′ | NPTII gene | CCACAGTCGATGAATCCAGAAAAG | PCR, confirming T-DNA insert |
| TDNA-1 |  | TAGACGGTTTTTCGCCCTTTGACG | TAIL |
| TDNA-2 |  | CGATTTCGGAACCACCATCAAACAGGA | TAIL |
| TDNA-3 |  | AACTCTCTCAGGGCCAGGCGGTGAAG | TAIL |
| OXT6-1 | At1g30460 | GGATCCATTAGGCCATTGAGCACAGCACT | Cloning for complementation |
| OXT6-2 | At1g30460 | CTGCAGACAACTTCAAGCGCAATCTGGTAA | Cloning for complementation |
| OXT6-3 | At1g30460 | ATGGAGGATGCTGATGGACTT | Small cDNA cloning |
| OXT6-4 | At1g30460 | CAGAACCCAATTAAAAACCTTAG | Small cDNA cloning |
| OXT6-5 | At1g30460 | ATGGAGGATGCTGATGGACTT | Large cDNA cloning |
| OXT6-6 | At1g30460 | CCGACGCTTCTTTCCTTCAC | Large cDNA cloning |
| OXT6-7 | At1g30460 | GGACTCTGAACACACCTATTTAC | Probe for Northern, RT-PCR (5′ end) |
| OXT6-8 | At1g30460 | GGTACTGGTCCTCTGAAATC | Probe for Northern, RT-PCR (3′ end) |
| OXT6-9 | At1g30460 | GAATTAGAGCCCTCCGTTGGT | Probe for Northern, RT-PCR (3′ end) |
| OXT6-10 | At1g30460 | CTCTTCTTCTCGTTTTGCTTCTGC | RT-PCR (3′ end) |
| Met-5′-1 | At3g09390 | ATGTCTTGCTGTGGAGGAAACTGC | 3′-RACE |
| Met-5′-2 | At3g09390 | GTGTGATCCTTGCACCTGCAAGTGA**A** | 3′-RACE |
| Thion-5′-1 | At5g36910 | ATGGAAGGCAAAACTGTGATCTCAAGTC | 3′-RACE |
| Thion-5′-2 | At5g36910 | CTTCTGTCTGCACCGGAGGATCTA | 3′-RACE |
| E2-5′-1 | At1g64230 | ATGGCTTCGAAAAGGATCTTGAAAGAG | 3′-RACE |
| E2-5′-2 | At1g64230 | GAGCAAAGTATGAGTCTACTGCGAGAAGCT | 3′-RACE |
| 3′RACE RT primer |  | GAGAGATGAAGTGAACTTGCGGGGTTTTTTTTTTTTTTTTTTVN | RACE-RT |
| 3′ RACE nested primer |  | GAGAGATGAAGTGAACTTGCGGGGTT | RACE - PCR |
| 5′ | At5g06690 | ATGGCATTTGAAAACTTAAAAATATCTCGTCAC | Microarray confirmation cloning |
| 3′ | At5g06690 | CTAGACAAATTTGTTGATCAATTCTCTAACTTC | Microarray confirmation cloning |
| plasmids |  | Plasmid designation |  |
|  | At5g04260 | U18029 | Microarray confirmation |
|  | At3g25580 | U60922 | Microarray confirmation |
|  | At1g07960 | U50593 | Microarray confirmation |
